# Dried Loquat Fruit Extract Containing Chlorogenic Acid Prevents Depressive-like Behaviors Induced by Repeated Corticosteroid Injections in Mice

**DOI:** 10.3390/molecules28145612

**Published:** 2023-07-24

**Authors:** Dong Wook Lim, Guijae Yoo, Changho Lee

**Affiliations:** Division of Functional Food Research, Korea Food Research Institute, Wanju 55365, Republic of Korea; dwlim@kfri.re.kr (D.W.L.); gjyoo@kfri.re.kr (G.Y.)

**Keywords:** *Eriobotrya japonica* fruit, chlorogenic acid, corticosterone, depression, anxiety, in vivo study

## Abstract

*Eriobotrya japonica* (loquat tree) has been used in traditional medicine to treat respiratory ailments, inflammation, and skin diseases; however, its potential antidepressant-like effects have not been extensively investigated. In this study, we evaluated the antidepressant-like effects of *E. japonica* fruit extract (EJFE) in a mouse model of corticosterone (CORT)-induced depression. An HPLC analysis revealed that chlorogenic acid (CGA) is the major compound in EJFE. Male ICR mice (5weeks-old) were injected with CORT (40 mg/kg, intraperitoneally) once daily for 21 days to induce depressive-like behaviors. Various behavioral tests, including the open field test, rotarod test, elevated plus maze (EPM), passive avoidance test (PAT), tail suspension test (TST), and forced swim test (FST), were conducted 1 h after the oral administration of EJFE at different doses (30, 100, and 300 mg/kg) and CGA (30 mg/kg). High-dose EJFE and CGA significantly alleviated CORT-induced depressive-like behaviors, as indicated by the reduced immobility times in the TST and FST. A decrease in the step-through latency time in the PAT, without an effect on locomotor activity, suggested an improvement in cognitive function. Moreover, EJFE- and CGA-treated mice exhibited significantly reduced anxiety-like behaviors in the EPM. Our results imply the promising potential of EJFE containing CGA as a therapeutic candidate for depression.

## 1. Introduction

The prevalence of depression on a global scale surpasses 300 million individuals, thus representing a significant concern for public health [1]. The efficacy of currently available first-line antidepressants may be limited, and they can also give rise to unfavorable side effects [2] Alternative therapeutic approaches, such as the use of herbs like *Hypericum perforatum* (St. John’s Wort) [3] or increased consumption of antioxidant-rich fruits and vegetables, have gained attention as potential treatments for depression [4]. The effectiveness of St. John’s wort and antidepressants in treating depressed patients was found to be similar according to Association for the Treatment of Depression (APA) guidelines [5]. Therefore, identification of novel antidepressants derived from natural sources, with high efficacy and minimal side effects, is pertinent.

*Eriobotrya japonica*, also known as the loquat tree, is a plant species native to southeastern China and is cultivated worldwide for its edible fruit [6]. *E. japonica* fruit is a rich source of dietary fiber, low in fat, and contains most of the essential minerals. The fruit is often processed into jams, jellies, and juices [7]. *E. japonica* has been used in traditional medicine to treat respiratory ailments, such as cough and bronchitis, inflammation, as well as skin diseases [8]. Various parts of *E. japonica*, including its leaves, fruits, seeds, and bark, have been investigated for their pharmacological effects [7]. Several studies have reported the antioxidant [9], anti-inflammatory [10], hypoglycemic [11], anti-obesity [12], and hepatoprotective [13] properties of *E. japonica*. Recently, the leaf extract of *E. japonica* was reported to exert neuroprotective effects on mice with β-amyloid-induced memory impairment by downregulating the Aβ1-42 peptide [14]. Moreover, polysaccharides derived from the leaf extract of *E. japonica* demonstrated protective effects in a mouse model of ischemic reperfusion, mediated through their antioxidant and anti-inflammatory properties [15]. These pharmacological effects are associated with the high polyphenol content of *E. japonica*, highlighting its potential as a therapeutic agent for neurologic diseases [16]. However, previous studies have not elucidated the antidepressant effects of *E. japonica* fruit extract (EJFE). In this study, we aimed to confirm the antidepressant effect of EJFE and its major compound, chlorogenic acid (CGA), in a mouse model of corticosterone (CORT)-induced depressive-like behavior [17]. The effect of EJFE and CGA on depressive behaviors in mice was assessed using various behavioral tests including the open-field test (OFT), rotarod test, elevated plus maze (EPM), passive avoidance test (PAT), tail suspension test (TST), and forced swim test (FST).

## 2. Results

### 2.1. Effect of EJFE and CGA in Tail Suspension Test (TST) and Forced Swim Test (FST)

**Figure 1:**
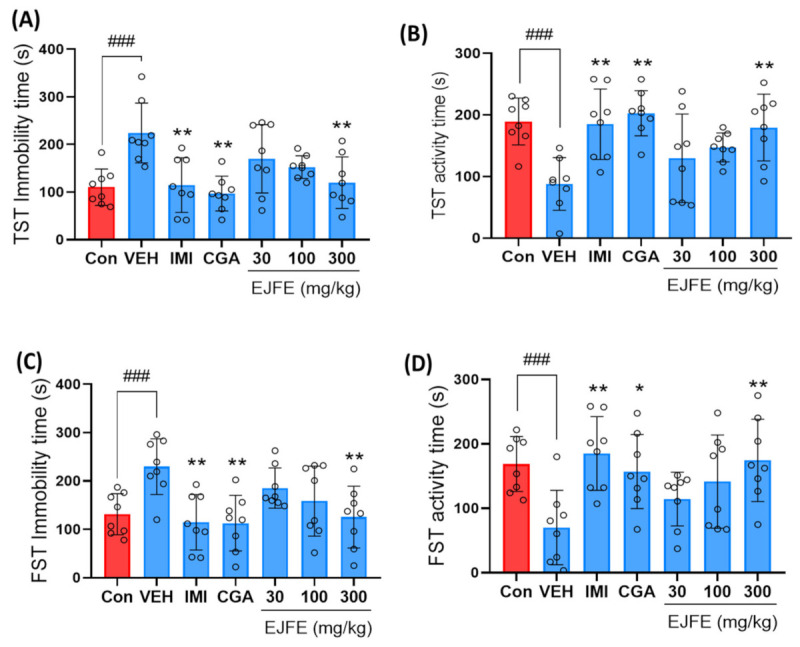
Effect of EJFE and CGA on CORT-induced depressive mice during TST and FST. CORT-injected mice exhibited significantly increased immobility and decreased activity, including swimming, while mice treated with EJFE at doses of 300 mg/kg showed significant improvements, with decreased immobility (**A**,**C**) times (s) as well as increased activity (**B**) and swimming (**D**) times (s). Results are presented as mean ± SD (n = 8, per group). Differences among experimental groups were determined via analysis of variance (ANOVA). ### *p* < 0.001 versus the Con group; * *p* < 0.05 and ** *p* < 0.01 versus the CORT-injected VEH group. Con, normal; VEH, vehicle; CORT, corticosterone; IMI, imipramine; CGA, chlorogenic acid; EJFE, *E. japonica* fruit extract.

Immobility observed during TST or FST is a representative behavioral indicator of a depressive-like phenotype in rodent models. In our study, CORT-injected mice demonstrated significantly increased immobility and decreased activity than the normal mice. However, IMI treatment significantly improved depressive-like symptoms in mice. The increased immobility caused by CORT treatment was significantly reduced by EJFE and CGA treatments (Figure 1A,C). Moreover, increased activity was observed in mice in the EJFE and CGA treatment groups (Figure 1B,D). These results indicate that EJFE and CGA treatments induced antidepressant-like effects in our mouse model of CORT-induced depression.

### 2.2. Effect of EJFE and CGA in Open Field Test (OFT)

**Figure 2:**
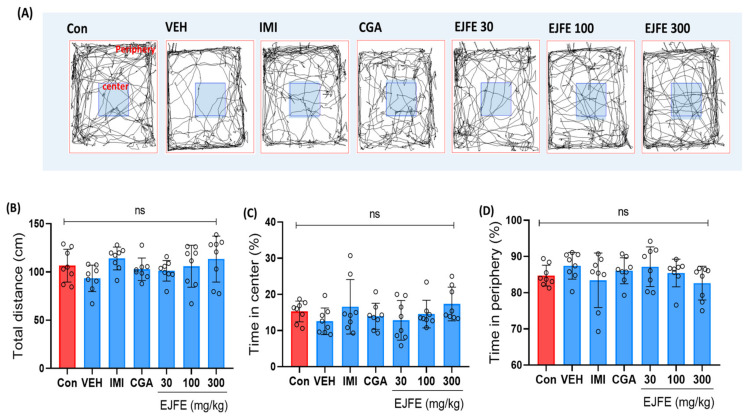
Effect of EJFE and CGA on CORT-induced depressive mice during OFT. A representative trace of locomotor activity during the 5-min observation period in OFT (**A**). Total distance (cm) traveled during OFT (**B**). The number of line crossings at the center of the field (**C**). The number of line crossings in the periphery of the field (**D**). No significant differences were observed among the groups in terms of locomotor activity. Results are presented as the mean ± SD (n = 8, per group). Differences among experimental groups were determined via analysis of variance (ANOVA). ns, not significant; Con, normal; VEH, vehicle; CORT, corticosterone; OFT, open-field test; EPM, elevated plus maze; PAT, passive avoidance test; TST, tail suspension test; FST, forced swim test; IMI, imipramine 30 mg/kg; CGA, chlorogenic acid 30 mg/kg; EJFE, *E. japonica* fruit extract.

We performed OFT to investigate the effect of EJFE and CGA on CORT-induced depressive-like behavior in mice. In previous studies, no differences in the locomotor activity were observed between CORT-injected and non-CORT-injected mice [18]. Based on the representative tracing results, the total distance traveled by mice in the vehicle-treated group was slightly lower than that of mice in the control group; moreover, a slight but statistically insignificant difference was observed in the center or corner retention rates (Figure 2A). Our findings revealed no significant differences among the groups for time spent in the center or peripheral area and the total distance traveled during OFT (Figure 2B–D).

### 2.3. Effect of EJFE and CGA in Rotarod Test

**Figure 3:**
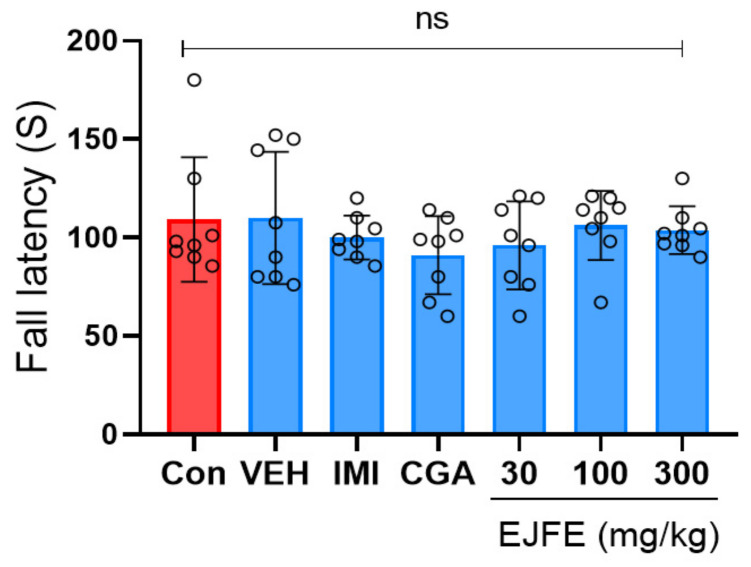
Effect of EJFE and CGA on CORT-induced depressive behavior in mice in the rotarod test. No significant behavioral alternations with regard to motor coordination were observed among the treatment groups. Results are presented as mean ± SD (n = 8, per group). Differences among experimental groups were determined via analysis of variance (ANOVA). ns, not significant; Con, normal; VEH, vehicle; CORT, corticosterone; IMI, imipramine; CGA, chlorogenic acid; EJFE, *E. japonica* fruit extract.

The rotarod test was conducted to investigate the effects of EJFE and CGA on motor coordination in mice with CORT-induced depression. As depicted in Figure 3, no difference in fall latency (s) was observed between the CORT-injected vehicle and normal mice. Similarly, the IMI-, CGA-, and EJFE-treated mice exhibited no significant changes in motor coordination.

### 2.4. Effect of EJFE and CGA in Elevated Plus Maze (EPM) Test

**Figure 4:**
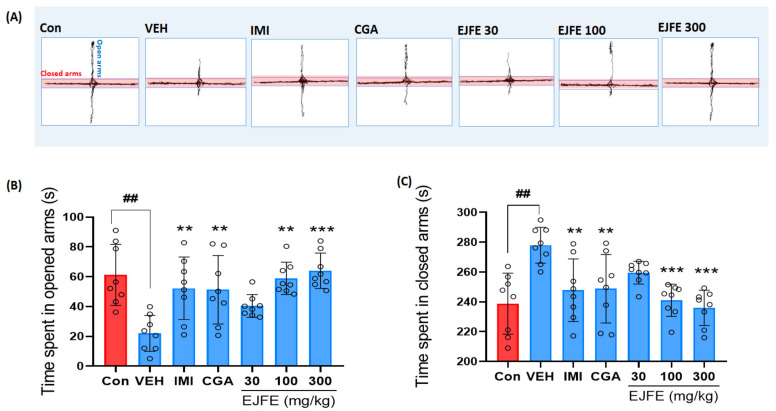
Effect of EJFE on CORT-induced depressive mice in the EPM test. A representative trace of locomotor activity during the 5-min observation period in EPM (**A**). Mice in the EJFE treatment group showed a significant improvement in CORT-induced anxiety-like behavior, as indicated by the significantly more time spent in the open arms (**B**) and less time in the closed arms (**C**). Results are presented as mean ± SD (n = 8, per group). Differences among experimental groups were determined via analysis of variance (ANOVA). ## *p* < 0.01 versus the Con group; ** *p* < 0.01 and *** *p* < 0.001 versus the CORT-injected VEH group. Con, normal; VEH, vehicle; CORT, corticosterone; IMI, imipramine; CGA, chlorogenic acid; EJFE, *E. japonica* fruit extract.

We conducted an EPM test, one of the most widely used methods for measuring anxiety-like behaviors in mice. In the EPM test, compared with the normal mice, those with stress-induced anxiety preferred movements in the closed arms and exhibited significantly fewer movements in the open arms [19]. Furthermore, CORT-injected vehicle mice spent significantly less time in the open arms and more time in the closed arms. Notably, mice in the CGA 30 mg/kg and EJFE treatment groups showed a significant improvement in CORT-induced anxiety-like behavior, particularly at a dose of 300 mg/kg (Figure 4B,C).

### 2.5. Effect of EJFE and CGA in Passive Avoidance Test (PAT)

**Figure 5:**
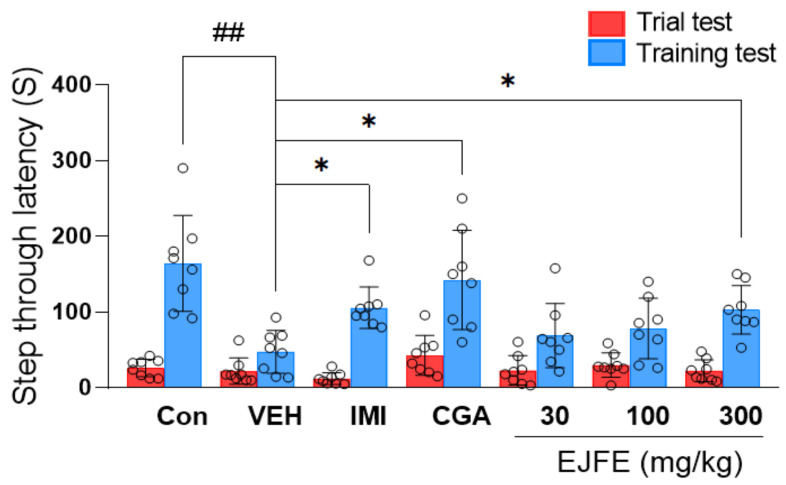
Effect of EJFE on CORT-induced depressive mice in PAT. CORT-injected vehicle mice exhibited a significantly decreased step-through latency time (s), whereas administration of EJFE at doses of 300 mg/kg significantly increased the latency time. Results are presented as mean ± SD (n = 8, per group). Differences among experimental groups were determined via analysis of variance (ANOVA). ## *p* < 0.01 versus the Con group; * *p* < 0.05 versus the CORT-injected VEH group. Con, normal; VEH, vehicle; CORT, corticosterone; IMI, imipramine; CGA, chlorogenic acid; EJFE, *E. japonica* fruit extract.

To investigate the effect of EJFE on CORT-induced cognitive dysfunction, a depressive symptom, we conducted a PAT. Previous research has demonstrated that mice treated with IMI exhibit decreased step-through latency time, implying a reversal of CORT-induced depressive behavior in these mice, consistent with the findings of our study [20]. Additionally, we observed that EJFE and CGA treatments significantly alleviated CORT-induced memory loss, suggesting that EJFE and CGA may alleviate depressive-like behaviors, including memory deficits, in mice treated with CORT (Figure 5).

## 3. Discussion

To the best our knowledge, this is the first study to examine the antidepressant-like effects of EJFE on mice with CORT-induced depressive-like behaviors. EJFE-treated mice exhibited a reversal of depressive-like behaviors, as indicated by significantly decreased immobility times in the TST and FST. Further, EJFE improved cognitive function by reducing the step-through latency time in the PAT without affecting locomotor activity when compared to that of sham mice. EJFE-treated mice also showed a significant improvement in CORT-induced anxiety-like behavior in the EPM.

Despite an incomplete understanding of the complex pathogenesis of depression, hypothalamic–pituitary–adrenal (HPA) axis dysfunction is an established risk factor for stress-related disorders, including depression or anxiety [21]. The association between HPA axis dysfunction and depression is supported by the observation that numerous patients with depression exhibit cortisol hypersecretion [22] and an impaired negative feedback of the glucocorticoid system [23]. In vivo model studies have also demonstrated that HPA axis dysfunction is exacerbated by chronic stress and is controlled by antidepressant treatment [24,25]. Furthermore, excessive exposure to glucocorticoids through repeated high-dose injections of CORT has been implicated in the development of depressive-like behaviors [26]. Mice that were repeatedly injected with CORT, especially at a dose of 40 mg/kg, exhibit depressive-like behaviors, as evidenced by an increase in immobility time in the FST or TST, without an effect on locomotor activity [27,28,29]. It has been reported that the state of immobility in the FST or TST mimics depression phenotypes in humans and is improved with antidepressant drugs [30]. Similarly, we found that mice injected with 40 mg/kg CORT daily exhibited significantly increased immobility times in the TST and FST. Meanwhile, mice treated with EJFE showed significantly reduced immobility times and increased activity time in the TST and FST, without any changes in locomotor activity, especially at a dose of 300 mg/kg. These results suggest that EJFE exerts antidepressant-like effects in an animal model of CORT-induced depression.

Cognitive impairment is a core feature of depression, and its symptoms are common among depressed patients [31]. Cognitive impairment is also observed in mice with CORT-induced depression [20]. Lee et al. reported that the injection of excessive CORT results in a significant impairment of neuronal function characterized by memory and cognitive deficits [32]. Animal behavioral experiments are widely used in the study of cognitive impairment, and the PAT is a representative behavioral experiment for evaluating improvements in cognitive function [33]. As expected, we found that mice in the CORT-treated control group exhibited cognitive impairment, indicated by a decrease in step-through latency time in the PAT, as opposed to the significant increase in step-through latency time observed in EJFE-treated mice, thus suggesting an improvement in cognitive function.

Depression is highly comorbid with anxiety disorders [34], with both animal and human studies reporting the anxiolytic effects of antidepressants [35]. The EPM test is a widely used behavioral assay for rodents that has been validated for the assessment of anxiolytic pharmacological agents and the study of mechanisms underlying anxiety-related behavior [36]. Increased time spent in the open arms indicated a lower degree of anxiety in the animal [37]. Our EPM results indicate that repeated injections of CORT resulted in anxiety-like behavior in mice, with significantly less time spent in the open arms and more time in the closed arms. However, the group of mice treated with EJFE showed a significant improvement in CORT-induced anxiety-like behavior, particularly at a dose of 300 mg/kg. These results suggest that EJFE has anxiolytic effects on CORT-induced anxiety-like behaviors in mice.

Phenolic compounds are secondary metabolites that are widely found in fruits, with flavonoids and phenolic acids as the most prominent examples. Research on dietary fruit phytochemicals has largely focused on phenolic compounds, owing to their diverse activities [38]. Neochlorogenic acid, chlorogenic acid (CGA), caffeic acid, and ellagic acid have been identified in E. japonica fruits [39]. These compounds have been shown to possess high antioxidant capacities, exerting beneficial effects in a range of oxidative stress-related diseases, including depression [40]. Through HPLC analysis, we identified chlorogenic acid as a major component of EJFE.

CGA was reported to exhibit antidepressant-like effects in a stress hormone-induced depressive animal model through inhibition of monoamine oxidase B (MAOB) activity and reactive oxygen species (ROS) production [41]. It also exerted neuroprotective effects in a 1-methyl-4-phenyl-1,2,3,6-tetrahydropyridine (MPTP)-induced Parkinson’s disease model by inhibiting mitochondrial dysfunction-mediated neuronal apoptosis [42]. It is therefore plausible that CGA is the active compound responsible for EJFE’s antidepressant effects, and our animal behavior experiments are consistent with this hypothesis. This is supported by the significant improvement in depressive-like behavior observed in the CORT-induced depression mice model following CGA treatment. However, further research should be conducted to determine EJFE’s efficacy against depression and the roles of different active compounds present within, in addition to studies on the exact mechanism of action.

## 4. Materials and Methods

### 4.1. Sample Preparation

**Figure 6:**
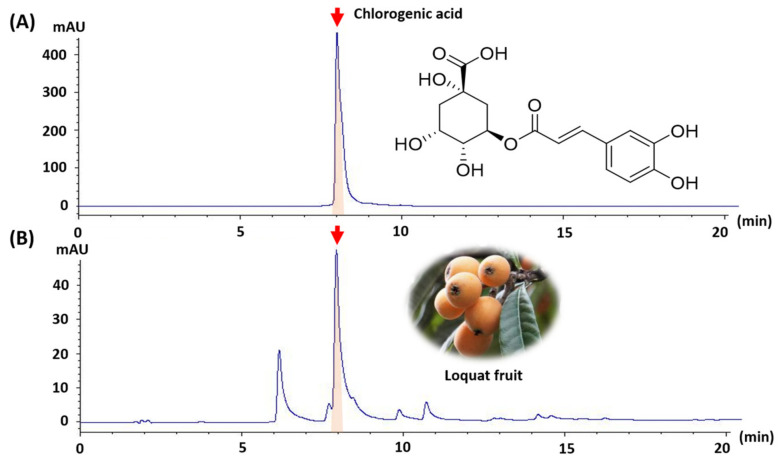
HPLC chromatogram of (**A**) chlorogenic acid as a standard compound and the (**B**) *E. japonica* fruit extract (EJFE). Chlorogenic acid peak was indicated by red arrow. The concentration of chlorogenic acid was 5.33 ± 0.08 mg/g EJFE.

Dried *E. japonica* fruit was purchased from the Jecheon medicinal plant market (Jecheon, Republic of Korea) and subjected to extraction using ethanol (70%, *v*/*v*) for 6 h at 70 °C using a reflux device. Thereafter, the EJFE was lyophilized and stored in a refrigerator until further use in experiments. The freeze-dried yield of the extract was 23.48% (*w*/*w*). EJFE was injected (5 μL) and analyzed via HPLC (Agilent Technologies, CA, USA) with diode array detector (DAD, 320 nm) using a Waters XBridge C18 (4.6 × 150 mm, 3.5 µm) column. The mobile phase was performed by gradient elution using 0.1% formic acid (A) and acetic acid (B). The solvent (A) was decreased from 90% to 5% in 35 min; the flow rate was 1 mL/min. The concentration of chlorogenic acid (CGA) was 5.33 ± 0.08 (mean ± SD) mg/g (Figure 6).

### 4.2. Animals and Treatments

**Figure 7:**
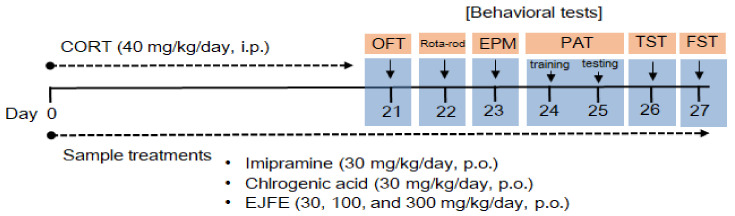
Behavioral experiment design in an animal model of depression induced by repeated CORT administration. CORT, corticosterone; OFT, open-field test; EPM, elevated plus maze; PAT, passive avoidance test; TST, tail suspension test; FST, forced swim test; IMI, Imipramine; CGA, chlorogenic acid; EJFE, *E. japonica* fruit extract.

Male ICR mice (5-weeks-old, 21–25 g) were obtained from KOATECH Animal Inc. (Pyeongtaek, Republic of Korea) and housed in accordance with the guidelines of the Institutional Animal Care and Use Committee (IACUC) of the Korea Food Research Institute (KFRI-M-19016). The mice were maintained under a 12 h light–dark cycle in a temperature-controlled environment at 21 ± 2 °C. All mice underwent an adaptation period of at least 1 week before the experiment. Based on our previous report [20], depression-like behaviors were induced in the mice through repeated intraperitoneal (i.p.) injections of CORT (40 mg/kg) for three weeks. The mice were randomly classified into seven groups (n = 8 per group): (1) Normal (Control, Con), (2) CORT + vehicle (CORT + VEH), (3) CORT + imipramine 30 mg/kg (CORT+IMI), (4) CORT + chlorogenic acid 30 mg/kg (CORT + CGA30), (5) CORT + EJFE 30 mg/kg (CORT + EJFE30), (6) CORT + EJFE 100 mg/kg (CORT + EJFE100), and (7) CORT + EJFE 300 mg/kg (CORT+RMFE300). All samples were orally administered once daily, and CORT was administered 1 h after oral administration of the samples. The behavioral experiments were conducted 1 h after CORT injection, as per the experimental scheme (Figure 7). Mice in the Con group received the same volume of vehicle.

### 4.3. Open Field Test

The OFT is commonly used in studies on depression and anxiety-like behaviors in rodents [43]. The locomotor activity of the mice were measured in the open field maze for 5 min. The total moving distance (cm) and time rate of center and peripheral zones (%) were analyzed by SMART software (SMART v3.0, Panlab SL, Barcelona, Spain).

### 4.4. Rotarod Test

The Rotarod test was performed as previously described. Each mouse was placed on a rotarod (Ugo Basile, Varese, Italy) accelerating from 1 to 30 rpm, and the latency time before falling was measured for 300 s.

### 4.5. Elevated Plus Test

The EPM is one of the most widely used tests for measuring anxiety-like behaviors in mice [44]. Mice were placed in the central zone of the maze at a height of 50 cm and allowed to explore the maze freely. The mice were placed in the central sector and were allowed to freely explore the maze. Mice behaviors were recorded for 10 min and analyzed using SMART software (SMART v3.0, Panlab SL, Barcelona, Spain).

### 4.6. Passive Avoidance Test

The PAT was performed using the passive avoidance device (GEMINI, SD instruments, San Diego, CA, USA), as previously described [45]. Each mouse was placed in a bright safe zone and allowed to acclimate for 1 min, then allowed to enter the dark zone through an automatically opened middle door. When the mouse entered the dark area, an electric shock of 0.5 mA was applied for 3 s. The next day, the latency time from the safe zone to the dark zone was recorded using the same experimental method.

### 4.7. Tail Suspension Test

The TST is widely used to screen potential antidepressant agents [46]. Mice were suspended using adhesive tape on hooks that automatically measured mice movements. The immobility time of the mouse was automatically measured for a total of 6 min through the automated TST apparatus (BioSeb, Chaville, France).

### 4.8. Forced Swin Test

The FST is one of the most commonly used tests for studying depressive-like behaviors in rodents. The potential anti-depressant-like effects of EJFE was verified using the FST, which was performed as previously described [28]. The mice were placed in a cylinder of water at a depth of 10 cm (22–24 °C), and their free-moving behavior was video-recorded for 6 min. Immobility and accommodation times were analyzed for the last 4 min out of a total of 6 min using SMART version 3.0 software.

### 4.9. Statistical Analysis

Data are expressed as mean ± standard deviation, and statistical significance was analyzed using one-way analysis of variance (ANOVA) by Prism 8 (GraphPad Software v8.0, Inc., San Diego, CA, USA). Statistical significance was set at *p* < 0.05.

## 5. Conclusions

Our findings strongly indicate that EJFE containing CGA ameliorates stress hormone-induced depressive and anxiety-like behaviors, as indicated by the significantly decreased immobility times in the TST and FST, as well as the improved cognitive function with reduced step-through latency time in the PAT, without effects on locomotor activity. Therefore, EJFE may represent a promising candidate for the treatment of depression.

## Data Availability

The data presented in this study are available on request from the corresponding authors.

## References

[1] Ali A.M., Alkhamees A.A., Hori H., Kim Y., Kunugi H. (2021). The Depression Anxiety Stress Scale 21: Development and Validation of the Depression Anxiety Stress Scale 8-Item in Psychiatric Patients and the General Public for Easier Mental Health Measurement in a Post COVID-19 World. Int. J. Environ. Res. Public Health.

[2] Racagni G., Popoli M. (2010). The pharmacological properties of antidepressants. Int. Clin. Psychopharmacol..

[3] Pirotta M., Willis K., Carter M., Forsdike K., Newton D., Gunn J. (2014). ‘Less like a drug than a drug’: The use of St John’s wort among people who self-identify as having depression and/or anxiety symptoms. Complement. Ther. Med..

[4] Liu X.Q., Yan Y., Li F., Zhang D.F. (2016). Fruit and vegetable consumption and the risk of depression: A meta-analysis. Nutrition.

[5] Guideline Development Panel for the Treatment of Depressive Disorders (2021). Summary of the clinical practice guideline for the treatment of depression across three age cohorts. Am. Psychol..

[6] Zhang Y.Y., Zhou Q., Rather L.J., Li Q. (2021). Agricultural waste of *Eriobotrya japonica* L. (Loquat) seeds and flora leaves as source of natural dye and bio-mordant for coloration and bio-functional finishing of wool textile. Ind. Crops Prod..

[7] Dhiman A., Suhag R., Thakur D., Gupta V., Prabhakar P.K. (2022). Current Status of Loquat (*Eriobotrya japonica* Lindl.): Bioactive Functions, Preservation Approaches, and Processed Products. Food Rev. Int..

[8] Li E.N., Luo J.G., Kong L.Y. (2009). Qualitative and Quantitative Determination of Seven Triterpene Acids in *Eriobotrya japonica* Lindl. by High-Performance Liquid Chromatography with Photodiode Array Detection and Mass Spectrometry. Phytochem. Anal..

[9] Jung H.A., Park J.C., Chung H.Y., Kim J., Choi J.S. (1999). Antioxidant flavonoids and chlorogenic acid from the leaves of *Eriobotrya japonica*. Arch. Pharm. Res..

[10] Kim T.M., Paudel K.R., Kim D.W. (2020). *Eriobotrya japonica* leaf extract attenuates airway inflammation in ovalbumin-induced mice model of asthma. J. Ethnopharmacol..

[11] De Tommasi N., De Simone F., Cirino G., Cicala C., Pizza C. (1991). Hypoglycemic effects of sesquiterpene glycosides and polyhydroxylated triterpenoids of *Eriobotrya japonica*. Planta Med..

[12] Sharma B.R., Oh J., Kim H.A., Kim Y.J., Jeong K.S., Rhyu D.Y. (2015). Anti-Obesity Effects of the Mixture of *Eriobotrya japonica* and *Nelumbo nucifera* in Adipocytes and High-Fat Diet-Induced Obese Mice. Am. J. Chin. Med..

[13] Nishioka Y., Yoshioka S., Kusunose M., Cui T.L., Hamada A., Ono M., Miyamura M., Kyotani S. (2002). Effects of extract derived from *Eriobotrya japonica* on liver function improvement in rats. Biol. Pharm. Bull..

[14] Kim M.J., Lee J., Seong A.R., Lee Y.H., Kim Y.J., Baek H.Y., Kim Y.J., Jun W.J., Yoon H.G. (2011). Neuroprotective effects of *Eriobotrya japonica* against beta-amyloid-induced oxidative stress and memory impairment. Food Chem. Toxicol..

[15] Huang X., Hou R., Pan W., Wu D., Zhao W., Li Q. (2022). A functional polysaccharide from *Eriobotrya japonica* relieves myocardial ischemia injury via anti-oxidative and anti-inflammatory effects. Food Funct..

[16] Liu Y., Zhang W., Xu C., Li X. (2016). Biological Activities of Extracts from Loquat (*Eriobotrya japonica* Lindl.): A Review. Int. J. Mol. Sci..

[17] Zhao Y., Ma R., Shen J., Su H., Xing D., Du L. (2008). A mouse model of depression induced by repeated corticosterone injections. Eur. J. Pharmacol..

[18] Sturm M., Becker A., Schroeder A., Bilkei-Gorzo A., Zimmer A. (2015). Effect of chronic corticosterone application on depression-like behavior in C57BL/6N and C57BL/6J mice. Genes Brain Behav..

[19] Hata T., Nishikawa H., Itoh E., Funakami Y. (2001). Anxiety-like behavior in elevated plus-maze tests in repeatedly cold-stressed mice. Jpn. J. Pharmacol..

[20] Lim D.W., Park J., Jung J., Kim S.H., Um M.Y., Yoon M., Kim Y.T., Han D., Lee C., Lee J. (2020). Dicaffeoylquinic acids alleviate memory loss via reduction of oxidative stress in stress-hormone-induced depressive mice. Pharmacol. Res..

[21] Mello A.F., Mello M.F., Carpenter L.L., Price L.H. (2003). Update on stress and depression: The role of the hypothalamic-pituitary-adrenal (HPA) axis. Braz. J. Psychiatry.

[22] Schule C., Baghai T.C., Eser D., Rupprecht R. (2009). Hypothalamic-pituitary-adrenocortical system dysregulation and new treatment strategies in depression. Expert. Rev. Neurother..

[23] Mason B.L., Pariante C.M. (2006). The effects of antidepressants on the hypothalamic-pituitary-adrenal axis. Drug News Perspect..

[24] Raone A., Cassanelli A., Scheggi S., Rauggi R., Danielli B., De Montis M.G. (2007). Hypothalamus-pituitary-adrenal modifications consequent to chronic stress exposure in an experimental model of depression in rats. Neuroscience.

[25] Szymanska M., Budziszewska B., Basta-Kaim A., Jaworska-Feil L., Kubera M., Regulska M., Leskiewicz M., Lason W. (2008). The effect of antidepressant drugs on the hypothalamic-pituitary-adrenal axis regulation in an animal model of depression. Bipolar Disord..

[26] Iijima M., Ito A., Kurosu S., Chaki S. (2010). Pharmacological characterization of repeated corticosterone injection-induced depression model in rats. Brain Res..

[27] Marks W., Fournier N.M., Kalynchuk L.E. (2009). Repeated exposure to corticosterone increases depression-like behavior in two different versions of the forced swim test without altering nonspecific locomotor activity or muscle strength. Physiol. Behav..

[28] Lim D.W., Han T., Um M.Y., Yoon M., Kim T.E., Kim Y.T., Han D., Lee J., Lee C.H. (2019). Administration of Asian Herb Bennet (*Geum japonicum*) Extract Reverses Depressive-Like Behaviors in Mouse Model of Depression Induced by Corticosterone. Nutrients.

[29] Li Y.C., Liu Y.M., Shen J.D., Chen J.J., Pei Y.Y., Fang X.Y. (2016). Resveratrol Ameliorates the Depressive-Like Behaviors and Metabolic Abnormalities Induced by Chronic Corticosterone Injection. Molecules.

[30] Renard C.E., Dailly E., David D.J.P., Hascoet M., Bourin M. (2003). Monoamine metabolism changes following the mouse forced swimming test but not the tail suspension test. Fundam. Clin. Pharmacol..

[31] Atique-Ur-Rehman H., Neill J.C. (2019). Cognitive dysfunction in major depression: From assessment to novel therapies. Pharmacol. Ther..

[32] Lee B., Sur B., Shim I., Lee H., Hahm D.H. (2014). Baicalin improves chronic corticosterone-induced learning and memory deficits via the enhancement of impaired hippocampal brain-derived neurotrophic factor and cAMP response element-binding protein expression in the rat. J. Nat. Med..

[33] Kruk-Slomka M., Biala G. (2021). Cannabidiol Attenuates MK-801-Induced Cognitive Symptoms of Schizophrenia in the Passive Avoidance Test in Mice. Molecules.

[34] Manes S., Nodop S., Altmann U., Gawlytta R., Dinger U., Dymel W., Ehrenthal J.C., Joraschky P., Nolting B., Petrowski K. (2016). Social anxiety as a potential mediator of the association between attachment and depression. J. Affect. Disord..

[35] Borsini F., Podhorna J., Marazziti D. (2002). Do animal models of anxiety predict anxiolytic-like effects of antidepressants?. Psychopharmacology.

[36] Walf A.A., Frye C.A. (2007). The use of the elevated plus maze as an assay of anxiety-related behavior in rodents. Nat. Protoc..

[37] Carola V., D’Olimpio F., Brunamonti E., Mangia F., Renzi P. (2002). Evaluation of the elevated plus-maze and open-field tests for the assessment of anxiety-related behaviour in inbred mice. Behav. Brain Res..

[38] Haminiuk C.W.I., Maciel G.M., Plata-Oviedo M.S.V., Peralta R.M. (2012). Phenolic compounds in fruits—An overview. Int. J. Food Sci. Technol..

[39] Xu H.X., Li X.Y., Chen J.W. (2014). Comparison of phenolic compound contents and antioxidant capacities of loquat (*Eriobotrya japonica* Lindl.) fruits. Food Sci. Biotechnol..

[40] Godos J., Castellano S., Ray S., Grosso G., Galvano F. (2018). Dietary Polyphenol Intake and Depression: Results from the Mediterranean Healthy Eating, Lifestyle and Aging (MEAL) Study. Molecules.

[41] Lim D.W., Han T., Jung J., Song Y., Um M.Y., Yoon M., Kim Y.T., Cho S., Kim I.H., Han D. (2018). Chlorogenic Acid from Hawthorn Berry (*Crataegus pinnatifida* Fruit) Prevents Stress Hormone-Induced Depressive Behavior, through Monoamine Oxidase B-Reactive Oxygen Species Signaling in Hippocampal Astrocytes of Mice. Mol. Nutr. Food Res..

[42] Singh S.S., Rai S.N., Birla H., Zahra W., Rathore A.S., Dilnashin H., Singh R., Singh S.P. (2020). Neuroprotective Effect of Chlorogenic Acid on Mitochondrial Dysfunction-Mediated Apoptotic Death of DA Neurons in a Parkinsonian Mouse Model. Oxid. Med. Cell. Longev..

[43] Xu F., Peng D., Tao C., Yin D., Kou J., Zhu D., Yu B. (2011). Anti-depression effects of Danggui-Shaoyao-San, a fixed combination of Traditional Chinese Medicine, on depression model in mice and rats. Phytomedicine.

[44] Komada M., Takao K., Miyakawa T. (2008). Elevated plus maze for mice. J. Vis. Exp..

[45] Lim D.W., Han D., Lee C. (2022). Pedicularis resupinata Extract Prevents Depressive-like Behavior in Repeated Corticosterone-Induced Depression in Mice: A Preliminary Study. Molecules.

[46] Mitchell N.C., Gould G.G., Smolik C.M., Koek W., Daws L.C. (2013). Antidepressant-like drug effects in juvenile and adolescent mice in the tail suspension test: Relationship with hippocampal serotonin and norepinephrine transporter expression and function. Front. Pharmacol..

